# A school of public health responds to the pandemic: A case study from Washington D.C.

**DOI:** 10.3389/fpubh.2022.896195

**Published:** 2022-10-25

**Authors:** Adnan A. Hyder, Jane H. Thorpe, Eugene Migliaccio, Natasha Kazeem, Lynn R. Goldman

**Affiliations:** Milken Institute School of Public Health, The George Washington University, Washington, DC, United States

**Keywords:** COVID-19, pandemic, public health policy, higher education, remote learning

## Abstract

The emergence of COVID-19 immediately affected higher education, and the closure of campuses at the start of the pandemic in March of 2020 forced educational institutions to quickly adapt to changing circumstances. Schools of public health faced challenges not only of shifting to remote learning and work environments, but also uniquely redirecting public health research and service efforts toward COVID-19. This paper offers a case study of how the Milken Institute School of Public Health at the George Washington University (GWSPH), the only school of public health in the nation's capital, initially adapted to the COVID-19 pandemic. Using a modified version of the Public Health Preparedness and Response Core Competency Model created by the Association of Schools and Programs of Public Health and the Centers for Disease Control and Prevention, we analyze how GWSPH worked in three areas—research, education, service/operations. We reviewed this initial response across four domains: model leadership; communication and management of information; planning and improving practice; and protecting worker (and student) health and safety. The adaptation of the model and the analysis of GWSPH's initial response to the pandemic can be useful to other schools of public health and health sciences in the United States and beyond, in preparing for all hazards. We hope that such analysis also informs the current concerns of schools such as return to in-person education as well as planning for future public health crises.

## Introduction

The emergence of the novel coronavirus, known as COVID-19, was declared a public health emergency on 30 January 2020 and a pandemic on 11 March 2020 by the World Health Organization (WHO) (1). Person-to-person transmission facilitated the spread of the virus worldwide, with more than 247,472,724 confirmed cases and 5,012,337 deaths worldwide by 3 November 2021 according to the WHO (1). In the United States alone, there are over 46,100,447 confirmed cases and 746,705 deaths; while the District of Columbia, Maryland, and Virginia (DMV) have counted over 1,557,420 cases and 26,158 deaths by 3 November 2021 according to the Centers for Disease Control and Prevention (2, 3). The scale of this pandemic prompted a swift response from the medical and public health communities, including schools of public health.

On 3 February 2020, the government of the United States (U.S.) declared COVID-19 to be a public health emergency, and on 13 March 2020, a national public health emergency was declared under the Stafford Act (4). As part of this declaration, all state, local, territorial, and tribal partners became immediately eligible for the U.S. Federal Emergency Management Assistance (FEMA) public assistance program, which provides direct and financial assistance for emergency protective measures (5). By 22 April 2020, the U.S. President had approved major disaster declaration requests for all 50 states, the District of Columbia, Puerto Rico, the Virgin Islands, Guam, American Samoa, and the Commonwealth of the Northern Mariana Islands. Furthermore, FEMA's operational tempo had increased dramatically by July 2020 with the following situational awareness: 114 concurrent major disaster declarations, at least one in every state, in five territories, the Seminole tribe of Florida, and the District of Columbia (6).

Schools of public health have been a part of the academic enterprise in the United States for decades. The Association of Schools and Programs of Public Health (ASPPH) was created in 2013 and currently represents 193 institutions, 131 of which are also accredited by the Council on Education for Public Health (CEPH) (7, 8). The mission of the ASPPH is to strengthen the capacity of members by advancing leadership, excellence, and collaboration for academic public health. Graduates from institutions which are a part of the ASPPH are equipped with the knowledge to handle public health issues including global health and epidemics, environmental health and risks, nutrition and obesity, emergency services and natural disasters, health disparities (9). These schools have been affected in critical ways as a result of the COVID-19 pandemic from ensuring a transition to remote learning and responding to the demand for COVID-19 research and data to serving as subject matter experts in their own institutions to help safeguard their communities.

The Milken Institute School of Public Health of the George Washington University (GWSPH) was established in 1997 and is the only school of public health in Washington, DC (10). GWSPH houses seven departments, including: Biostatistics and Bioinformatics, Environmental and Occupational Health, Epidemiology, Exercise and Nutrition Sciences, Global Health, Health Policy and Management, and Prevention and Community Health. Across these departments GWSPH offers 25 masters level programs (including MPH, MS, MHA, joint degrees and online programs), 8 doctoral level programs (including PhD and DrPH), and three majors and four minors for undergraduate students. Unique to GWSPH is the MPH/PA joint degree program, the first in the country allowing students to pursue public health and Physician Assistant degrees at the same time. All academic programs are accredited by organizations like CEPH or the Commission on Accreditation of Healthcare Management Education (CAHME). Annual enrollment at GWSPH is roughly 2,300 graduate students and 575 undergraduate majors in a diverse student body (11).

GWSPH also affords students the opportunity to take part in applied practical exposures to gain experience working with public health professionals from a variety of disciplines. Such placements can include governmental entities, non-governmental organizations (non-profits), hospitals, private companies, and start-ups; allowing students to apply knowledge gained from coursework to real-world public health scenarios while gaining practical skills. GWSPH also has a core mission as a high performing, research-intensive, school of public health under the highest standards of research. It is actively engaged in multidisciplinary scholarship, hundreds of research projects conducted by research faculty with support of a dedicated Office of Research Excellence. All students have the opportunity to become involved with research projects and share their accomplishments through a university-wide research day.

In light of the COVID-19 pandemic and the goals of schools of public health in the United States, this paper aims to present a case study of how a school of public health responded to, and was integral to, the pandemic response. This case study focuses only on a set of responses to the pandemic and explores how GWSPH adjusted to the pandemic across key domains of academic functioning. We focus on the initial set of responses by the school, and analyze them using an existing ASPPH/CDC framework for preparedness, and reflect on key lessons. We hope that such a case study will not only help other schools of public health, but also schools in health sciences and medicine, to prepare for future crisis situations in the country and around the world.

## Conceptual framework

In 2010, the ASPPH and the CDC created the Public Health Preparedness and Response Core Competency Model, with the goal of training public health students to “*proficiently perform assigned prevention, preparedness, response, and recovery role(s) in accordance with established national, state, and local health security and public health policies, laws, and systems*” (12). This model includes four action-based competencies that both the ASPPH and CDC deemed necessary for emergency public health response: model leadership; communicate and manage information; plan for and improve practice; and protect worker health and safety (12, 13). The model also includes knowledge, skills, and attitudes that are necessary not only for holistic education of public health students but also integral in pandemic and emergency response situations.

**Figure 1:**
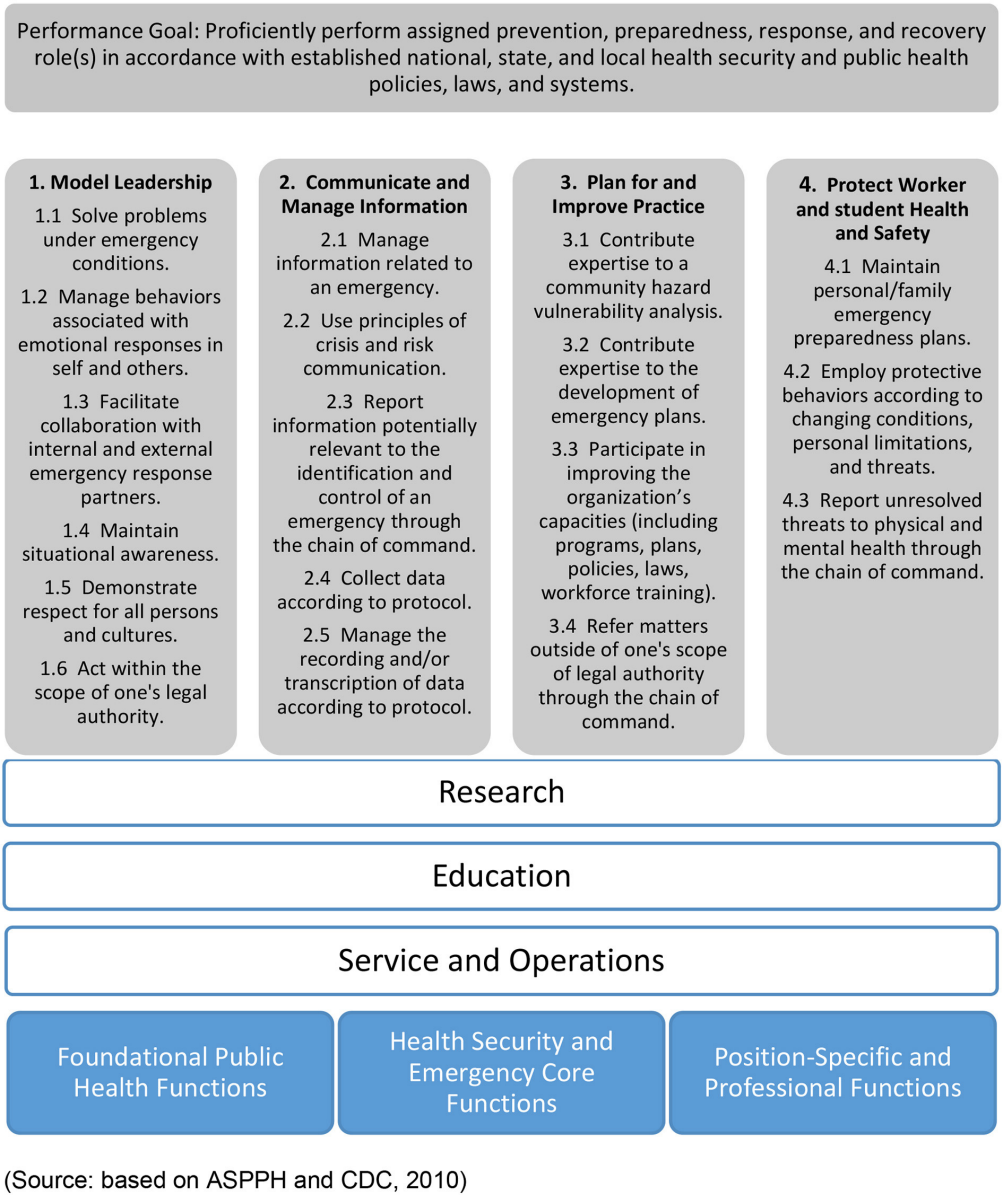
Modified framework for public health response model.

We believe this model provides a basic approach for institutional public health preparedness. We have adapted the model and proposed modifications (Figure 1) to focus not only on preparedness for public health emergencies, but also on guiding a response to such situations as well. The revised framework includes the four original competencies that encompass knowledge, skills, and attitudes but we made the following modifications: we edited the four competencies to suit pandemic responsiveness; so the content of the 18 competencies listed are now focused on preparedness. We added three cross cutting functions (research, education, and service/operations) common to most schools of public health to segment responses to the pandemic; and we removed the original three functions and integrated them into research, education, and service/operations. By applying the four original goals of the preparedness model to these integral areas of action, the framework can inform appropriate actions that schools of public health can take to aid their students, faculty, communities, and the public in times of emergency. And key to these changes is that instead of focusing only on protecting worker health and safety in the original model, our adaptation expands it to include student and staff health and safety as well. This framework provides a conceptual map to both analyze but also plan for pandemic preparedness in schools and potentially other organizations.

We have used this modified framework to organize organizational responses during the current pandemic. In the following section, we analyze the response of GWSPH across the three domains (education, research, and service/operations), identifying both short- and long-term actions, as well as how these actions address the competencies recommended.

## The response

Based on the actions GWSPH took to respond to the COVID-19 pandemic, we identified key actions across education, research, service/operations (Tables 1, 2).

**Table 1:** GWSPH responses to the COVID-19 pandemic (examples).

	**Short-term**		**Medium/long-term action**	
	**Response**	**Example**	**Response**	**Example**
**Education**	-Shift to remote learning to protect students and staff	-COVID-19 Webinar series	-Providing funding for students financially impacted by the pandemic	-GW Cares student assistance fund
	-Increased access to COVID-19 information	-COVID-19 Information hotline	-Increased efforts to educate on social and health disparities	-Improved core curriculum with stronger focus on disparities
	-Increase in intake of MPH specializations.	-Partnerships with Zoom, Webex, Blackboard	-Increased efforts to teach on the implications of COVID-19 and prevention measures.	-Expanded platforms to enhance virtual teaching innovations
		-Remote teaching workshops		
		-Expanded opportunities for virtual field experiences		
**Research**	-Increased assistance to antigen production	-COVID-19 research fund	-Collection of longitudinal data from COVID-19 patients to analyze antigens and help in development of vaccine	-COVID-19 Specimen Bank
	-Funding for projects that address telehealth, drugs, therapies, or treatments	-Revised ethics IRB guidelines	-Large number of COVID-19 projects initiated	-COVID-19 testing laboratory established (BSL3).
	-Generate rapid preliminary data	-Requirement for COVID-19 prevention strategies for research studies	-Research fund established by GWSPH for COVID-19 research	
	-Safeguarding data while working remotely	-COVID-19 research studies granted priority		
	-Protection of human subject participants			
**Service and operations**	-Providing more accessible testing to students, staff, and community members	-Drive Through Testing	-Mobilizing volunteers to assist greater DC area with pandemic response	-GW Health Volunteer Task Force
	-Ensuring essential workers have adequate PPE	-Inventory of PPE for staff	-GWSPH students providing service as part of practicum or PEs	-Large number of GWSPH students took performed practicum and PEs in areas related to the pandemic
	-Providing guidance on covid-19 prevention	-SPH Dean and other members were involved in university wide structures for coordinating covid-19 efforts		
		-Public Health Laboratory		

**Table 2:** The framework and GWSPH decisions—examples.

	**Model leadership**	**Communicate and manage information**	**Plan for and improve practice**	**Protect worker health and safety**
**Research**	-Coordinated response with university	-Town halls and virtual meetings	-Develop SOPs for protection of staff and participants	-Implementation of COVID guidelines in research
	-Establishment of rules of engagement based on evolving evidence	-Rapid issuance of memos and guidance for researchers	-Mandatory review of all research studies prior to reopening	-Protection of workers key criteria for approval
**Education**	-Partnered with health and medical leadership to create community public service announcements.	- Consistent communication and interactions with students and faculty	-Expanded models and guidance for instructional continuity	-Continuity of information and resources related to health and wellbeing
	-Coordinated academic response across degrees and programs	-Bi-weekly meetings with student organization leaders and all students		
		-Workshops for faculty		
**Service and operations**	-Appointed faculty leadership to serve framework to support community partners and train student volunteers	-Daily situation reports to support operations	-Built a DMV support model for placing hundreds of volunteers	-PPE inventory for all GWU essential workers
	-Faculty and staff leadership serving as SMEs in driving institutional-wide operational decisions informed by science	-Weekly team meetings	-Community engagement	-Developed testing protocols and return-to-work health policies
		-Volunteer support	-Volunteer Task Force procedure development	-Mandatory testing as well as vaccination.
		-COVID listserv and website creation	-Developed policies and procedures to influence long-term institutional policies.	
		-Creation of an internal EHR system through Point and Click		

SOP, standard operating procedures; PPE, personal protective devices; DMV, district of Columbia, Maryland, Virginia.

## Education

GWSPH made numerous shifts in teaching and learning to support students due to the COVID-19 pandemic. Short-term efforts included, first and foremost, the shift to remote learning for residential students to protect students, staff, and faculty health and safety by expanding the use of technologies such as Zoom©, Webex©, and Blackboard© and leveraging our experience and learning platforms from our online programs in partnership with vendors (such as 2U©) to ensure continuity of instruction. Frequent assessment of remote teaching opportunities and challenges were conducted using faculty surveys, faculty focused information sheets (in the form of Frequently Asked Questions—“FAQs”) with current academic information, and maintaining virtual teaching resources on shared online platforms. Spring, summer, and fall term faculty teaching workshops were also hosted to help support faculty transitions to online teaching.

Continuous engagement with students helped to understand the challenges they faced during the COVID-19 pandemic and how to best support them as well. Frequent assessment of remote learning opportunities and challenges were conducted using student surveys and student focused information sheets. GWSPH hosted bi-weekly meetings with student leaders and held open sessions for residential and online students every other week. Academic support policies were immediately implemented, allowing pass or no pass, and credit or no credit options for undergraduate and graduate students, as well as expanded access to existing online courses.

Experiential learning was moved online and departments began approving online experiences for the MPH practicum, culminating experience, and inter-professional experiences including an enhanced range of virtual opportunities to enable students to complete their coursework and experiential learning components on the timeline planned pre-COVID-19. Online students also were given the opportunity to participate in residential courses that were offered virtually (for the first time) during the pandemic as well.

The COVID-19 pandemic has also significantly changed long-term educational practices within GWSPH. Various funds have been established for students financially impacted by the pandemic, such as the “GW Cares”, a student assistance fund. Permanent changes have also been made to support inclusive teaching in virtual and in person classrooms, by developing and launching inclusive teaching resources in partnership with the GWSPH Master Teacher Academy. Classroom technologies have also been permanently expanded to support more flexible classrooms, such as integrating hybrid, virtual and in-person teaching styles when the opportunity to return to campus arises. Remote and in-person classroom technology orientation opportunities and remote teaching workshops to support faculty design classroom experiences in a hybrid setting were held.

Finally, special educational programs (such as the GWSPH Summer Institute; and other summer intensives) were rapidly converted from an on-campus to a fully online offering. While this required considerable energy, the advantage of extending the reach of these opportunities was impressive. This was also true for COVID-related webinars such as the “Ethics and COVID-19” webinar series started in April 2020 (and still running) that was able to reach an audience of thousands across not only the United States but also Africa, Asia and Latin America.

## Research

GWSPH took a series of measures to mitigate the effects of COVID-19 on research participants, projects, and outcomes. As early signs of the pandemic began to set in in early March 2020, GWSPH opted to continue research while recommending that investigators take steps to decrease the likelihood of spreading disease. All investigators were required to create actionable and concrete plans in the event that COVID-19 continued to spread and the university may shut down. If possible, data collection for face-to-face research changed to screen participants for flu-like symptoms or travel within the last 14 days, avoiding gathering groups of participants, conducting interviews over the internet, and following exposure guidelines. Just days later, as the situation continued to heighten, new guidance was issued instructing research teams, particularly clinical teams, to immediately screen all research participants for signs and symptoms of COVID-19 and assess how the disruption of active research protocols may harm participants.

During the following month of April 2020, all surrounding states—District of Columbia, Maryland, Virginia (DMV)—issued stay at home orders. At this time, GWSPH ensured either full transition of research studies to alternative (electronic) data collection methods or paused (or stopped) some studies. Principal investigators and research leads were encouraged to evaluate whether their work provided high direct benefit to participants while planning how to safely continue work in such unprecedented times.

By July, GWSPH established guidelines for research projects that needed to operate (or resume) based on four principles (and consistent with larger university guides): 1) priority for the health and safety of GWSPH faculty, staff and participant communities; 2) ensuring that science, evidence, and pragmatism guide decisions; 3) promoting flexibility and innovation in the face of evolving circumstances; and 4) striving to provide inclusive and equitable solutions for all research. All principal investigators were required to create and submit a detailed proposal for operations, which was expedited for review by the GWSPH Office of Research Excellence in concert with university offices.

By Fall 2020, research projects that wished to resume (or new studies) were required to submit a detailed plan with full COVID precautions for approval to the Office of Research Excellence (including approvals at the department level). The focused reorganization of all research work to ensure implementation of COVID protocols, provision of better support to researchers, and creation of more sustainable (virtual) models of research management were all achieved with strong support of GWSPH faculty and staff.

The university developed guidelines for marking the status of research which included the shutdown of activities due to COVID-19 (or initial response) where research was essentially online except critical studies and COVID-related research with only essential personnel observing all safety protocols (e.g., social distancing, personal hygiene, decontamination, PPE). A “limited” reopening starting the summer of 2020 meant strict monitoring of population density (e.g., 25% of lab capacity), rotation of team members, strict observation of all safety protocols, review of chemical or radiological hazards, review of graduate students and postdocs, review of grant timelines and donor rules, and training of all personnel. Later in the Fall of 2020 an “expanded” reopening involved review of research personnel (special permission needed to be on campus) including mandatory training, regular COVID-19 testing, and strict public health guidelines; enhanced population density (e.g., up to 50% of lab capacity); review of studies conducted off campus; and opening of core facilities (while adhering to all safety and density guidelines). A future phase of total resumption of research was also discussed with the hope of addressing the pandemic.

GWSPH followed university-wide guidelines for *laboratory-based research* including: developing a comprehensive list of all study or lab personnel with contact information and roles; accommodating social distancing requirements with plans for de-densification of personnel (determining bench/workspace to map adequate distance or physical separation, developing work shifts to ensure a safe environment for all personnel); documenting the types of experiments and activities within different parts of a lab; determining use of cubicles and shared offices; understanding the critical use of shared tissue culture and other spaces; documenting equipment, supplies and reagents that might need to be purchased; and estimating Personal Protective Equipment (PPE) usage in the labs (e.g., gloves, masks) as per university and DC policy.

Throughout this process GWSPH focused on necessary components to safe and effective conduct (or resumption) of research. This focus included attention to: appropriate methods to mitigate risk of infection or transmission of COVID-19 to, and among, research participants and staff to influence the risk/benefit ratio of research involving in-person interactions; appropriately informing research participants of the risks of COVID-19 related to research participation and of the COVID-19 infection mitigation strategies undertaken by the university and research team (this would also address the need for informed consent of prospective research participants); and prioritization and monitoring of research with university offices (e.g., Office of Human Subjects Research) to determine what types of research can be conducted (or resume). These measures were meant to ensure that all research was carried out in a manner that minimizes the chance of COVID-19 transmission between research participants and the study team; avoid overuse of space and school resources; and identify priority (especially COVID-19 related) research at that time.

Finally, during this time GWSPH also responded to the need for data on the COVID-19 pandemic itself with zeal. For example, between April 2020 and Feb 2021, near 70 COVID-related research proposals were submitted to donor agencies and nearly $4.5 million worth of external grants were awarded. Intramural funding (with the university) was also dedicated to innovative COVID-19 research and individual research centers (like the Center for AIDS Research, Fitzhugh Mullan Institute) mobilized their own resources and staff for COVID-related research (such as development of a health workforce calculator). Finally, GWSPH joined the university to host the first-even online research day to showcase studies across the school with a focus on student engaged work.

## Service and operations

A GWSPH COVID-19 task force was created to coordinate the GWSPH response and worked with health care systems and community-serving organizations across the DMV to identify shortcomings in care that volunteers can fill. Then, a Volunteer Task Force of over 400 members comprised of faculty, staff, and students with public health, clinical, and other health-related skills and expertise was organized. Such volunteers supported COVID-19 related work in local jurisdictions such as: departments of health in Maryland (Montgomery and Prince Georges counties), District of Columbia, and Virginia (Loudoun and Arlington counties); the National Association of County and City Health Officials (NACCHO); United Medical Center; DC Health Emergency Preparedness and Response Administration, and the DC-COVID-311 call center. This team provided contact tracing, education and training to new contact tracers; worked on contact tracing, case investigations, COVID-19 testing and general coordination; helped develop databases and database management tools; provided emergency operations support; and assisted at a variety of quarantine locations for the health departments. Moreover, GWSPH faculty served as primary points of contact and provided consultation for the organizations, created learning opportunities for students, and coordinated logistics for the volunteers.

GWSPH volunteers performed a variety of activities for example volunteers updated the NACCHO database to include COVID-19 information and contact information for each local health department within the United States, encompassing more than 3,500 locations. At the United Medical Center, volunteers assisted with the hiring of additional nursing assistant personnel to help with COVID-19 patients. GWSPH volunteers provided call center support *via* DC's 311 information line that included decreasing calls from concerned residents, triaging patients against a standard protocol, identifying sick patients that need to be transported to a healthcare facility, identifying infected patients that could be monitored or treated under isolation at home, and arranging for remote monitoring with home quarantined patients. Faculty volunteered their time to provide expert advice to the DC Mayor, NIH, and other agencies. Finally, onsite GWSPH faculty members also provided each student volunteer an important learning environment where they were able to apply their public health knowledge and skills to create safer communities during the COVID-19 pandemic.

Internally, initially using expertise and equipment that had been developed for performing NIH funded research, the GWSPH established a Public Health Laboratory (PHL) that developed a high-throughput largely automated polymerase chain reaction (PCR) test for COVID-19 to deploy for our campus community including essential workers. In the summer of 2020, it began routine testing for the university's designated on-site staff, which included tradespeople, housekeepers, law enforcement and environmental health and safety personnel. Upon receipt of an emergency use authorization (EUA) from the Food and Drug Administration (FDA) in August 2020, it began weekly testing that was required of all of the limited personnel who were authorized to be on campus. This group included students who are living on campus (~500) or those who had a class physically on campus (both in DC and in Ashburn, VA); and essential faculty, staff, and on-site contractors. The laboratory also ensured that positive results were communicated to either the occupational health and wellness unit or Colonial Health Center for clinical follow-up with the respective student, faculty, or staff member, as well as (by law) providing all results to DC and Virginia Health Agencies.

GWSPH also created the Campus COVID Support Team (CCST), a group led and staffed by GWSPH faculty and research staff; they were responsible for ensuring that all COVID-19 test results generated within the laboratory were immediately communicated to individuals. The CCST was also responsible for providing information about on campus contact tracing to D.C. and VA public health authorities and inform members of our community of the importance of cooperating with public health agencies in their contact tracing efforts. GWSPH epidemiology faculty developed practices for identifying and investigating COVID outbreaks; they and CCST also developed a COVID dashboard that provides daily updates about the results of testing.

The operations unit of GWSPH worked across all university offices to integrate signage for social distancing, directions to reduce foot traffic in high traffic areas, and cleaning or disinfection of spaces. It ensured that appropriate computer and online equipment was distributed to our community; and deployed user surveys to understand needs and facilitate triaging of issues *via* a helpdesk support function. It coordinated campus security and ensured building access contingent on compliance with university COVID testing. GWSPH also supported more immediate availability of COVID-19 information through their COVID-19 webinar series and the COVID-19 information hotline.

## Discussion

The COVID-19 pandemic has had an immense impact on education, particularly post-secondary education, and many institutions are looking to understand how the challenges created by the pandemic will impact them in the future. The list includes financial barriers, admissions and enrollment numbers, student support (including student loan payments, student work study, campus housing), and accommodations for international students (14). All these have also negatively impacted student performance. National and state policy makers in the United States have attempted to mitigate some of these burdens with legislation, such as the CARES Act and state-based policies, to appropriate funds toward education (15). However, such institutions still face a number of continuing expenses and the long-term impact of decisions such as hiring freezes, pay cuts, furloughs, and suspension of retirement plans (14).

Studies have shown that research outputs have been significantly impacted as a result of COVID-19 across academia. Institutions ranging from small college campuses to the National Institutes of Health have seen a financial impact, resulting in research output losses, depleted budgets, disinvestment, and the inability to reach initial goals (16). In order to remain a global leader in research and innovation, it is essential that higher education institutions in the United States continue to prioritize research and find new ways to revolutionize the research process during the pandemic.

Many hours of service and technical consultation were provided by faculty at the GWSPH and in schools and programs across the country. While this kind of activity is considered to be an essential component for faculty in public health (as clinical work is for medical school faculty), we find it is not consistently valued by external reviewers for promotion and tenure decisions. At GWSPH, we communicate to reviewers that we value impactful work in this area. Moreover, faculty up for promotion and/or tenure were given the option of extending “the clock” in consequence to delays that might be created by COVID (17).

There are a number of things schools of public health, including GWSPH, will need to implement to thrive in the post-pandemic world. It is expected that schools will continue maximizing online education platforms, not just as the pandemic evolves, but into the future. By training faculty, transferring tutoring, advising services, and administrative services online in addition to coursework, and developing strong customer relationships between technology departments and learning management systems, schools can strengthen their systems for long-term change. Other changes include a re-examination of admissions processes and criteria including the use of (or requirements for) standardized tests (18).

Managers and leaders will continue to face challenges of a hybrid workforce that is both virtual and in-person. Investing in environmental scans that solicit feedback from employees (faculty, staff) to help inform transition strategies is a critical component toward addressing morale challenges during this time of crisis. Having stronger emergency response efforts can help improve communication and morale and having strong systems in place can assist in the coordination of future efforts (19).

It is clear that a strong research system within an institution is important to support a school through, and out of, a pandemic (20, 21). This is an important learning from this case study—having great infrastructure, clear policies, electronic systems of review, and solid cohort of researchers allows an organization to withstand the financial and human resource challenges of an epidemic. This also means that developing both a talented group of public health researchers and an enabling environment (pre-award, post-award, ethics review, etc.) is critical during non-crisis times and ought to be a major investment area for the academy, especially schools of public health (22).

We used the modified framework presented above to organize the organizational responses during the early days of the current pandemic. The framework was useful to analyze the response of GWSPH across the three key academic domains (education, research, and service/operations), to identify short and long-term actions, as well as focus on key competencies that need to be addressed. In review future improvements in communication (especially to students), enforcement of safety rules, securing rapid testing at mass scale, and financial management have been planned. We hope these will help improve performance in the next scenario.

We can use these priority areas to continue to guide education, research, and service and operations during public health emergencies, and to enhance the utility of our framework guiding schools of public health (23–25). We hope that this case study can be useful for other schools as the COVID-19 pandemic progresses and institutions attempt to find their new normal. We also hope this case can guide schools in best practices as they plan for future semesters and for reopening beyond the current pandemic.

## Data availability statement

The original contributions presented in the study are included in the article/supplementary material, further inquiries can be directed to the corresponding author.

## Author contributions

All authors wrote sections of the manuscript and contributed to manuscript revision, read, and approved the submitted version.

## Funding

This work was partly supported by the Office of Research Excellence, Milken Institute School of Public Health, George Washington University, Washington DC, USA. The content is solely the responsibility of the authors and does not necessarily represent the official views of the George Washington University.

## Conflict of interest

The authors declare that the research was conducted in the absence of any commercial or financial relationships that could be construed as a potential conflict of interest.

## Publisher's note

All claims expressed in this article are solely those of the authors and do not necessarily represent those of their affiliated organizations, or those of the publisher, the editors and the reviewers. Any product that may be evaluated in this article, or claim that may be made by its manufacturer, is not guaranteed or endorsed by the publisher.
